# Genetic variability and population structure analysis of *Protostrongylus oryctolagi* (Nematoda: Protostrongylidae) in *Lepus europaeus* from Central and Northern Italy

**DOI:** 10.1371/journal.pone.0313998

**Published:** 2025-01-09

**Authors:** Ilaria Guarniero, Laura Stancampiano, Rafaella Franch, Elisa Armaroli, Fabio Macchioni, Enrico Negrisolo

**Affiliations:** 1 Department of Veterinary Medical Sciences, University of Bologna, Ozzano nell’Emilia, Bologna, Italy; 2 Department of Comparative Biomedicine and Food Science (BCA), University of Padova, Legnaro, Italy; 3 Studio Geco, Piazza Pighini, Arceto, Reggio Emilia, Italy; 4 Department of Veterinary Sciences, University of Pisa, Pisa, Italy; 5 Department of Agronomy, Food, Natural Resources, Animals and Environment (DAFNAE), University of Padova, Legnaro, Italy; University of Bari, ITALY

## Abstract

Nematodes are abundant and ubiquitous animals which are poorly known at intraspecific level. This work represents the first attempt to fill the gap on basic knowledge of genetic variability and differentiation in *Protostrongylus oryctolagi*, a nematode parasite of lagomorphs. 68 *cox1* sequences were obtained from brown hares collected in five locations in Northern and Central Italy, highlighting the presence of a high amount of genetic variation inside this species. The eleven haplotypes identified (Haplotype diversity equal to 0.702) were split into two lineages: lineage A (comprising six different haplotypes, A1-A6) and lineage B (B1-B5). The mean intra-lineage amount of genetic variation was 0.3%, whereas the inter-lineage percentage of variation was ten-fold higher (3%). These two lineages were non-randomly distributed in the investigated areas. Lineage A showed a preference for Central Italy (Tuscany) even if it was sporadically found also in northern territories (Emilia-Romagna), while B-haplotypes were present exclusively in Emilia-Romagna. The analysis of molecular variance identified two main barriers to gene flow: (i) a strong major one which separate samples of Central Italy (PIA and GR7) from the northern ones (RE1, RE3 and MO1; Φ_ST_ = 0.750, P = 0.00); (ii) a secondary faint barrier which separates Pianosa island from Grosseto (Φ_ST_ = 0.133, P = 0.00). Any difference was found among northern samples (Φ_ST_ = 0.009, P = 0.00). The observed data may be explained by several factors ranging from the parasite’s biology (presence of a narrow host spectrum), the final host’s behaviour (small home range), the natural dispersion of the host-parasite dyad occurred in past or the recent passive men-mediated migration. Finally, the presence of unconventional shortened amplicons revealed the presence of NUMTs (nuclear copy of mitochondrial genes) in the *P*. *oryctolagi* nuclear genome, suggesting caution when using DNA barcode as unique marker for the identification of species belonging to this genus.

“*In short*, *if all the matter in the universe except the nematodes were swept away*, *our world would still be dimly recognizable*”. Nathan Augustus Cobb, from "Nematodes and Their Relationships", 1915

## Introduction

“*In short*, *if all the matter in the universe except the nematodes were swept away*, *our world would still be dimly recognizable*”.

Nathan Augustus Cobb, from "Nematodes and Their Relationships", 1915

With more than 40,000 known species and 400 new taxa described annually, nematodes represent one of the most widely distributed, abundant, and diverse group of metazoans [1]. A significant part of them developed parasitic life-strategy in vertebrate definitive hosts [1]. Although parasite nematodes are a highly biodiverse clade, for a series of reasons spanning from their hidden nature, challenging sampling and, sometimes, difficult species identification, little is known about their intraspecific genetic variability. This knowledge gap should be filled with new studies since the understanding of population genetic structure of parasite nematodes has profound implications for understanding biodiversity and its evolution over time, revealing how populations respond to selective pressure [2], and to clarify some evolutionary questions like the development of parasitic lifestyle and host specialization [3].

Besides its value in increasing the basic knowledge of global biodiversity, studying the population genetic structure of nematode parasites has additional benefits, revealing ecological and biological aspects. Indeed, it is possible to increase our knowledge on parasites themselves, illuminating aspects of their biology (e.g. reproductive strategies, dispersal ability, infection dynamics), as well as their taxonomy (e.g. the presence of cryptic species or, conversely, of different morphotypes associated to not detectable genetic differentiation). Furthermore, it allows for a deeper understanding of their hosts, clarifying aspects of their diet, their dispersal ability and behaviour.

*Protostrongylus oryctolagi* Baboš, 1955 is a lung nematode parasite of lagomorphs with an indirect life cycle. The free-living first larval stage (L1) is released into the environment with the faeces of the definitive host. This larval stage soon moves into the intermediate gastropod hosts, where it moults to the L2 and L3 stages. The final host becomes infected by the ingestion of the L3 stage. After the ingestion, the L3 moves from the intestine to the lymphatic system, mutating to L4. Fourth stage larvae reach the lungs where they develop into adult males and females, and mate. The eggs laid by the females hatch in the lungs, L1 larvae pass through the pharynx and are released into the environment with the host faeces [4,5].

No data are available in literature about genetic variability and diversity of *P*. *oryctolagi*, being genetic markers used only for species identification [6].

The aim of this study is to analyse the genetic variability and differentiation of *P*. *oryctolagi* isolated from brown hares *Lepus europaeus* coming from Central and Northern Italy, investigating the possible drivers of parasite genetic structure.

## Materials and methods

### Sampling

Sixty-eight adult specimens of *P*. *oryctolagi* coming from 16 brown hares were collected between 2016 and 2020 in five different geographical areas (Table 1). Two areas were located in Central Italy, south of the Tusco-Emilian Apennines: Pianosa Island (PIA) and Grosseto province (GR7); the remaining three areas were located north of the Apennines, specifically two from Reggio Emilia and one from Modena provinces (RE1, RE3 and MO1 respectively). All the sampling areas are flat, except RE3 with a hilly environment. Areas RE1 and MO1 are subjected to a heavy agricultural pressure and thus present a highly homogeneous environment, with large fields and massive anthropization. All hares were legally shot during 2020 hunting season, except those from Pianosa island, which were collected as described in [7].

**Table 1:** Sampling details.

N	H	Collection Site	Sample code	Catch Period
21	5	Pianosa	PIA	Winter 2016^§^
15	2	Grosseto	GR7	Autumn 2020
12	3	Reggio Emilia	RE1	Autumn 2020
16	4	Reggio Emilia	RE3	Autumn 2020
4	2	Modena	MO1	Autumn 2020

N: Number of parasites sequenced. H: Number of different brown hares analysed; Collection Site: Geographical sampling area; Sample code: Except for Pianosa which is a protected area, all other codes correspond to the ATC (Ambito Territoriale di Caccia) code. ^§^ Hares from Pianosa were harvested as a part of the eradication plan of “LIFE13 NAT/IT/000471 project—RESTO CON LIFE” in 2016 winter season.

### DNA isolation, amplification, and sequencing

DNA was extracted from the middle-anterior part of each nematode by Wizard Genomic DNA Purification Kit (Promega) according to the company’s protocol and stored at -20°C for the downstream analyses. After morphological and molecular identification according to Guarniero et al. [7], the DNA barcode region of the mitochondrial gene encoding for the Cytochrome Oxidase I (*cox1*) protein was amplified using the primer couple HCO2198 / LCO1490 [8]. The PCR mix contained 50 ng of DNA, 10 pmol of each primer, 12,5 μl of DreamTaq Green PCR Master Mix (Thermo Scientific) and PCR-grade water up to 25μl of final reaction volume. The thermal profile consisted of 5 minutes at 94°C, followed by 30 cycles of 30 seconds at 94°C, 30” at 50°C and 30” at 72°C, followed by a final 7 minutes elongation step at 72°C. PCR products were purified by ReliaPrep DNA Clean-Up and Concentration System (Promega) and sequenced by StarSEQ facility (Germany).

### Bioinformatic analyses

The obtained *cox1* sequences were trimmed to encompass an exact number of codons. To perform molecular evolution and phylogenetic analyses, orthologous sequences of species belonging to the superfamily Metastrongyloidea [9] were included in the dataset. Specifically: *Protostrongylus rufescens*, *Orthostrongylus macrotis*, *Varestrongylus eleguneniensis* (Family Protostrongylidae); *Angiostrongylus cantonensis*, *Angiostrongylus mackerrasae* (Angiostrongylidae); *Metastrongylus pudendotectus*, *Metastrongylus salmi* (Metastrongylidae). The 68 sequences of *P*. *oryctologi*, plus the seven outgroup sequences, were aligned by ClustalW (option codon) algorithm [10] implemented in MEGA11 [11] to generate the multiple alignment *Poryctolagi*_ALN0. This alignment was 648 positions long and did not include indels. *Poryctolagi*_ALN0 spanned from base 67 to base 714 of the full-length *cox1* sequence of *P*. *rufescens*.

A subset named *Poryctolagi*_NTWK, containing only the 68 sequences of *P*. *oryctolagi*, was obtained from *Poryctolagi*_ALN0 to perform the intraspecific molecular comparisons and to create a network (see below). Finally, the different *cox1* haplotypes identified for *P*. *oryctolagi* (see results) were combined with the outgroups to generate the dataset *Poryctolagi*_PHYL, that was used in the phylogenetic analysis.

### Computation of sequence statistics and network analysis

Different DNA statistics were computed on *Poryctolagi*_NTWK set using the software DnaSP 6.12.03 [12] and MEGA11 [11]. Starting from the *Poryctolagi*_NTWK a median joining network [13] was created with the program PopART 1.7 [14].

### Phylogenetic analysis

Phylogenetic analyses were performed on *Poryctolagi*_PHYL according to the maximum likelihood method [15]. The best tree search analysis (1,000 replicates) was performed with the software IQTREE 2.2.2.6 [16]. The best fitting evolutionary model was identified with the ModelFinder algorithm [17] implemented in IQTREE. Ultrafast bootstrap (UFBoot; 10,000 replicates) [18] and SH-like approximate likelihood ratio test (SH-alrt; 1,000 replicates) [19] were computed to assess the statistical support to the best tree topology.

### Analysis of population structure

The Analysis of Molecular Variance (AMOVA) [20] and the estimation of the related fixation indexes Φ was performed using Arlequin 3.5.2.2 [21] to evaluate the possible occurrence of barriers to geneflow giving rise to population structuring in *P*. *oryctolagi* sampled in five areas of Central and Northern Italy. Initially, overall AMOVA without hierarchies was applied to assess differences among the five populations sampled in the five geographical areas. The same preliminary analysis was used to evaluate differences among the three northern populations (MO1, RE1 and RE3) and between the two central populations (PIA and GR7). The final AMOVA was carried out grouping the populations according to the preliminary analyses to obtain an overall hierarchical model explaining *P*. *oryctolagi* population structure.

## Results

**Fig 1:**
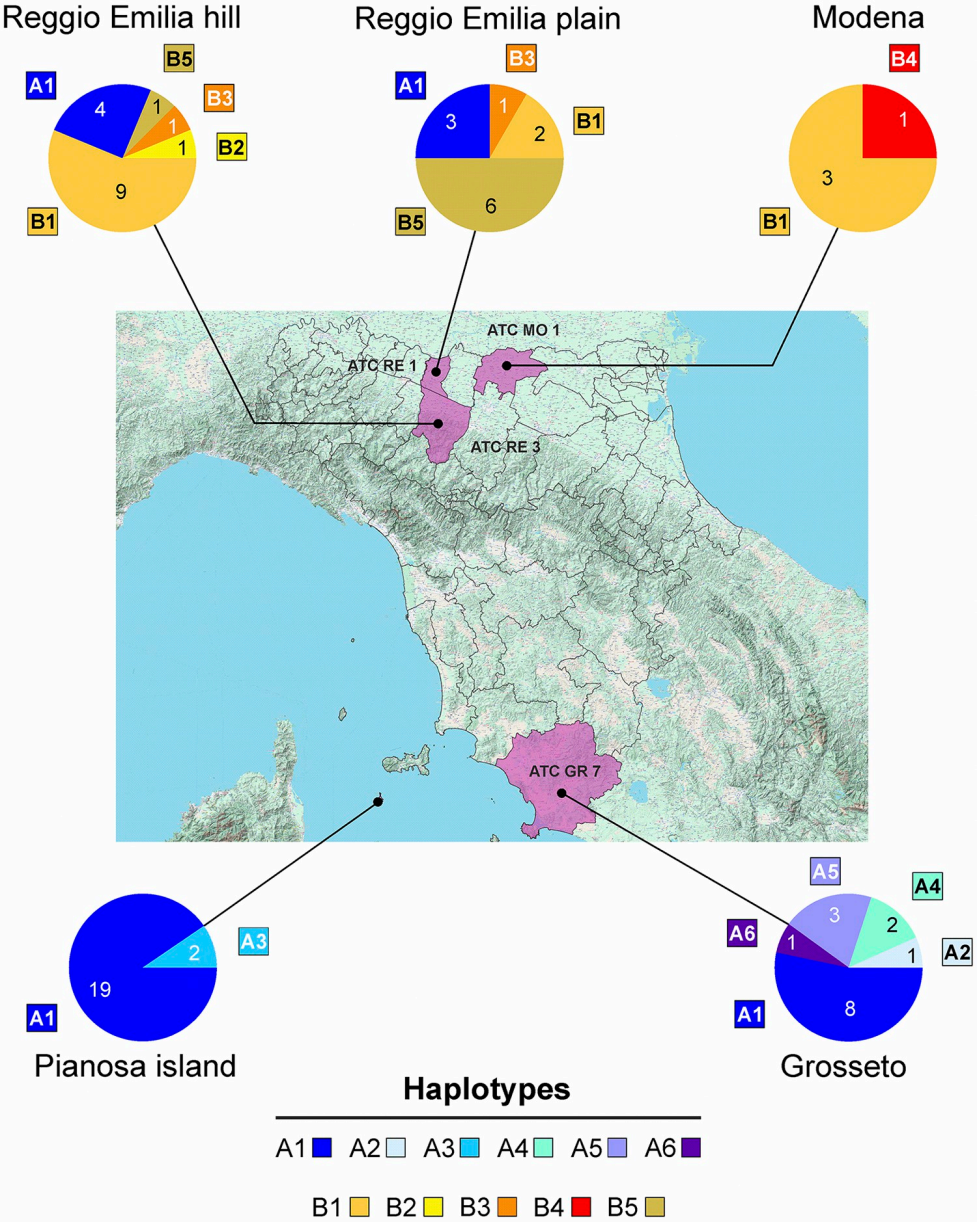
Haplotype distribution and frequency among different geographical sites.

The multiple alignment *Poryctolagi*_NTWK exhibited 26 variable sites (Figs 1 and S1), with nucleotide diversity per site π = 0.01473. Besides the standard 648 bp *cox1*-barcode sequence, in MO1_15_1 and MO1_19_1 specimens, we obtained a second shortened sequence of 591 bp that resulted to be a NUMT (nuclear copy of mitochondrial gene). The comparison between the NUMTs revealed a single nucleotide difference (A vs G, position 252; S2 Fig). The two NUMTs can be aligned unambiguously with the true *cox1* barcode counterparts, but they exhibit frameshifts, stop codons and deletions (S3 and S4 Figs). In the sequence of the NUMTs, portions of the *cox1* protein can still be detected, encoded in different reading frames (S3 and S4 Figs).

### Genetic variability

**Fig 2:**
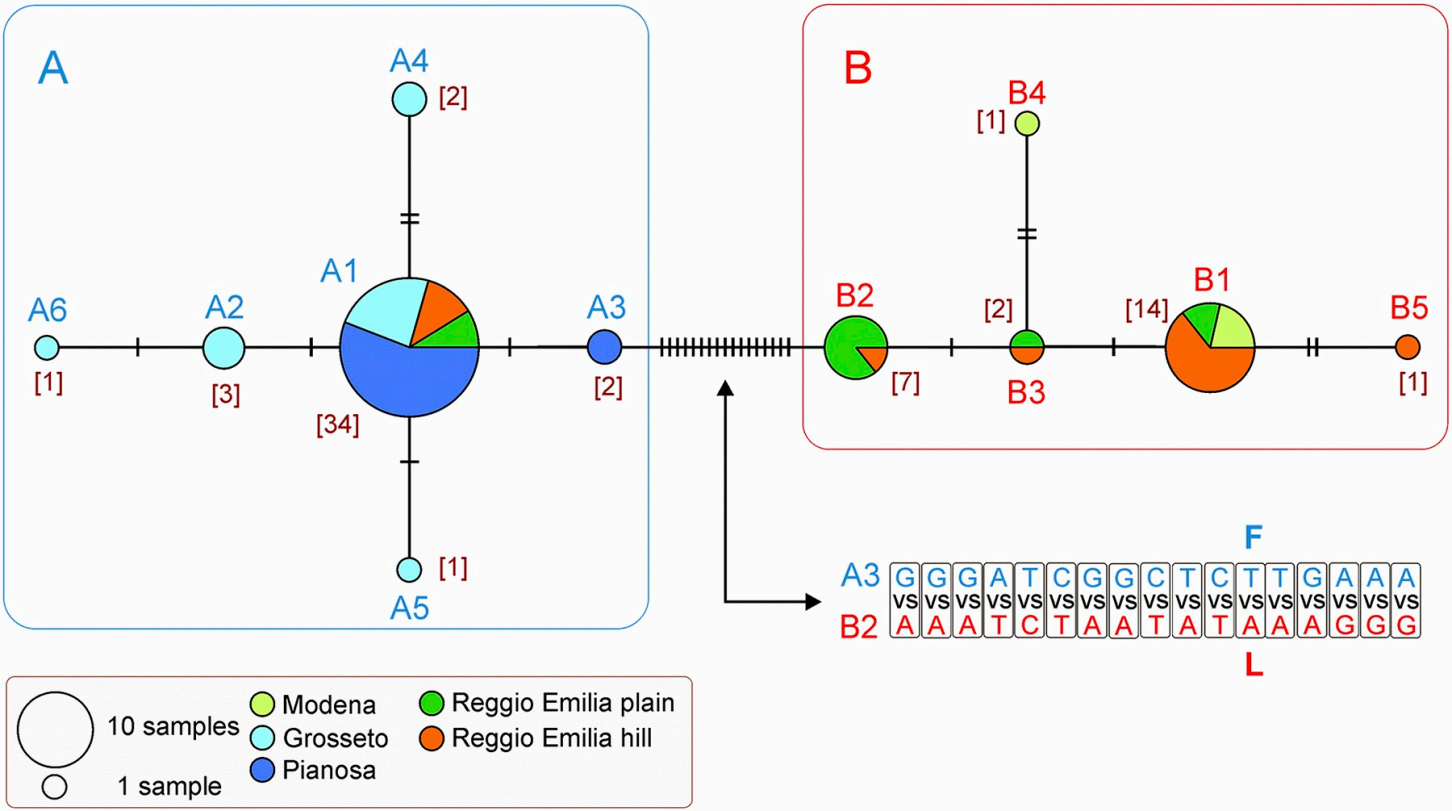
Median joining network produced with PopArt. A1-A6 and B1-B5, unique mitotypes belonging to group A and B. In square brackets are listed the number of sequences belonging to a peculiar mitotype. Double arrow points to the nucleotides and amino acid changes in the branch connecting A3 to B2. Vertical bar in a branch, one nucleotide change.

The median joining network (Fig 2) shows that the 68 sequences belong to 11 distinct mitochondrial haplotypes (or mitotypes; GenBank accession codes OR987131 to OR987141), with haplotype diversity Hd equal to 0.702. These mitotypes clustered in two distinct groups, group A (six mitotypes, A1-A6) and group B (five mitotypes, B1-B5). Twenty-three out of 26 variable sites are first- and mostly third-codon position, without amino acid changes (S1 Fig), while the remaining three represent each a different codon position resulting in a nonsynonymous aminoacidic translation. In particular, the T/C transition in position 98 (2^nd^ base codon; S1 Fig) determines a change of Val vs Ala in all mitotypes B except B2. The Ala amino acid is shared by all the A-mitotypes of *P*. *oryctolagi* as well as by the sequences of *Protostrongylus rufescens* and *Orthostrongylus macrotis* and therefore it is to be considered as background condition. The second nonsynonymous change (A/G transition in position 214, 1^st^ base codon; S1 Fig) implies the substitution Ser vs Gly in the mitotypes A2 and A6. Finally, the transversion A vs T in position 357 (3^rd^ base codon, S1 Fig) produces the change Phe vs Leu in all the mitotypes B, while the group A retains the protostrongylid amino acid plesiomorphic condition. The sequence variability within each group is very limited (see S1 Table). Specifically, the average p-distance is 0.003 ± 0.001 for A group and 0.004 ± 0.002 for B group. Conversely, the intergroup distance is ten-fold higher with an average value equal to 0.030 ± 0.002 (S1 Table). A minimum of 17 nucleotide changes are necessary to pass from the mitotypes belonging to group A to the mitotypes belonging to group B. The two most similar sequences between the two groups were A3 and B2 respectively (Figs 2 and S1 and S1 Table). Based on both network topology and molecular sequence comparisons, the two mitotype groups appear quite distinct.

### Phylogenetic tree of *P*. *oryctolagi* mitotypes

**Fig 3:**
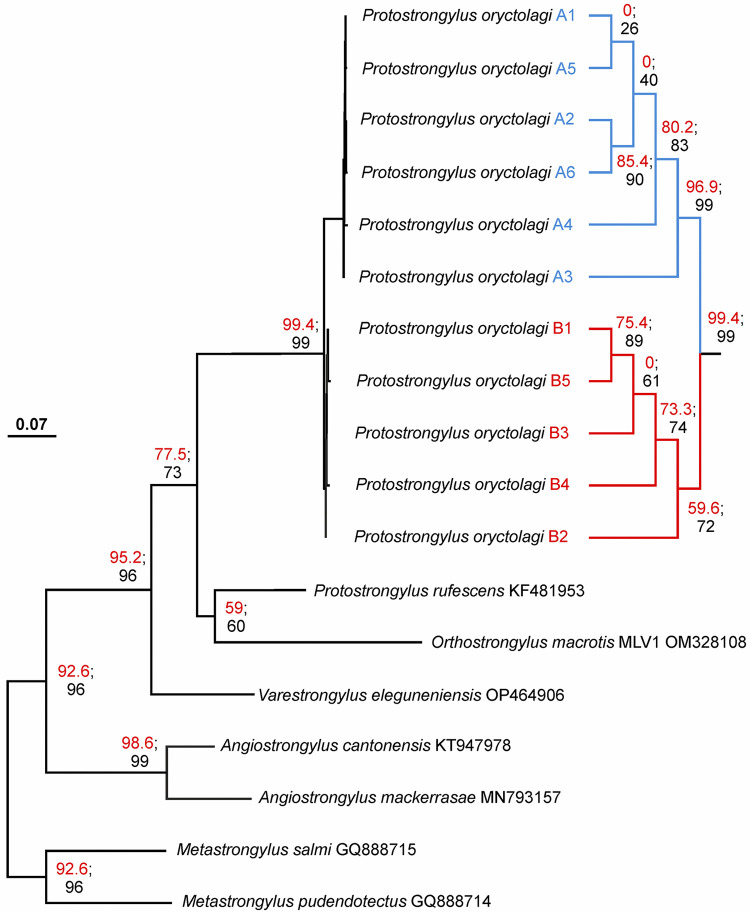
Phylogenetic relationships among unique mitotypes. Maximum likelihood tree (-log = 2659.5103) produced with IQTREE 2.2.2.6, by applying the best evolutionary model TIM+F+I+G4 selected by ModelFinder. In red, ultrafast bootstrap values, expressed in percentage. In black, SH-like approximate likelihood ratio test values expressed in percentage. Bar, number of substations for site.

The new mitotypes determined for *P*. *oryctolagi*, cluster in a very distinct monophyletic group receiving strong statistical corroboration. The tree topology confirms the presence of the two distinct mitotype groups A and B. The group A has strong statistical support (96.9% and 99%, see Fig 3), a behaviour not mirrored by group B. Furthermore, phylogenetic relationships within groups A-B are not well resolved. In the ML tree, *P*. *rufescens* is linked to *O*. *macrotis* instead to its congeneric *P*. *oryctolagi*. However, this relationship receives very limited statistical support.

### Genetic differentiation and population structure

Regarding the geographical distribution of mitotypes, *cox1* sequences belonging to group A were more widely distributed and found in *P*. *oryctolagi* specimens collected in all the sampled localities except MO1 (Fig 1). Specifically, the A1 mitotype was found thrice in RE1 sample and four times in RE3 (25% of representativeness in both cases). In the northern area, mitotype A was found in four hosts coinfected with parasites belonging to the B-group: a single hare coming from RE1 and three hares from RE3. In contrast, sequences of the B-group were limited to specimens recorded north of the Apennines (RE1, RE3, MO1; Fig 1). The overall AMOVA (Table 2A) highlighted an evident population structure, with significant differences among the five populations sampled and a very high fixation index (Φ_ST_ = 0.663, p<0.00001). No evidence of population structure was detected among the three northern populations (Table 2B), which were not significantly different with an overall fixation index Φ_ST_ next to zero (Φ_ST_ = 0.009, p = 0.294). Regarding the central areas (Table 2C), Pianosa island appeared slightly but significantly differentiated (Φ_ST_ = 0.133, p<0.00001) from the population coming from the mainland (GR7). Table 3 reports the final hierarchical AMOVA confirming that the northern populations (grouped to be compared with the other populations) were strongly differentiated from those of central Italy with high and highly significant fixation index (Φ_CT_ = 0.666, p<0.00001). The significant percentage of variation within populations (29.83%) is noticeable and indicates a good level of genetic variability (Table 3).

**Table 2:** AMOVA preliminary analyses.

**a**	**Overall, one group: PIA+GR7+MO1+RE1+RE3**			
*Source of variation*	*d*.*f*.	*Percentage of variation*	*Φ*_*ST*_	*significance*
Among populations	4	66.32	0.6632	p<0.00001
Within populations	63	33.68		
**b**	**north, one group: MO1+RE1+RE3**			
*Source of variation*	*d*.*f*.	*Percentage of variation*	*Φ*_*ST*_	*significance*
Among populations	2	0.86	0.0086	p = 0.294
Within populations	29	99.19		
**c**	**central, one group: PIA+GR7**			
*Source of variation*	*d*.*f*.	*Percentage of variation*	*Φ*_*ST*_	*significance*
Among populations	1	13.28	0.1328	p<0.00001
Within populations	34	86.72		

**Table 3:** AMOVA final model.

	Overall, three groups: PIA vs GR7 vs MO1+RE1+RE3						
*Source of variation*		*d*.*f*.	*Percentage of variation*	*Φc*_*T*_	*Φsc*	*Φs*_*T*_	*significance*
Among groups		2	66.55	0.6653			p<0.00001
Among populations within groups		2	3.62		0.1081		p = 0.327
Within populations		63	29.83			0.7017	p<0.00001

## Discussion

Population genetics surveys on nematode parasites of wild animals can aid in determining whether and how their intraspecific genetic divergence is geographically structured. These studies reveal the potential drivers of intraspecific genetic variability, like the presence of factors acting as homogenizer (e.g. presence of a broad host range and the long-distance dispersal behaviour of hosts) [1–3,22,23]. In addition to the presence of physical barriers, conditions of isolation, presence of a narrow host range and limited dispersal habits of the hosts are factors that may reduce gene flow [2].

In our study, the *cox1*-barcode has proved to be a handy tool for identifying population variability and structure in *P*. *oryctolagi* in five areas of Northern and Central Italy, bringing to light two different populations with different geographical distribution, the presence of barriers to gene flow and the related possible drivers that shaped the observed population structure.

### Overall amount of genetic variation and related drivers

The eleven mitotypes of *P*. *oryctolagi* here found, form a statistically strongly supported monophyletic group separated from all other reference sequences including the congeneric *P*. *rufescens* (Fig 3). This result suggests that the *cox1*-barcode sequence should be a reliable marker for defining the species boundaries in *P*. *oryctolagi*, as already proved for many nematodes taxa (e.g. [24–27]. However, this hypothesis must be tested and corroborated with an adequate screening of the diversity of Protostrongylidae family, which is currently lacking. Moreover, the discovery of NUMTs in the genome of *P*. *oryctolagi* suggests caution when using automated DNA-barcoding sequencing as unique tool for specimen identification especially in metabarcoding projects, to avoid misidentification of species as well as erroneous recognition of “new taxa” [28]. The occurrence of NUMTs in Nematoda is not a novelty and has already been observed in other parasite species like *Mansonella ozzardi* [29].

The eleven haplotypes revealed the presence of a substantial genetic variability, testified by the occurrence of eleven unique mitotypes split -genetically and, in some extent even geographically- into two main lineages. Lineage A is mainly present in Central Italy, while lineage B is exclusive of Northern Italy. The amount of genetic variation appears remarkably high if compared to the *cox1* sequence variation found in the nematode *Thelazia callipaeda* in dogs, foxes, and cats by Otranto et al. [30], who identified a unique haplotype distributed across Europe. Since the dispersal habit is one of the drivers, which promote the genetic homogenization of nematode parasite populations, the disparity observed between Otranto’s and our study could be ascribed to the different dispersal behaviour of mesocarnivore species versus lagomorphs. Considering hosts with similar or even smaller home range, a survey on the vector transmitted nematode *Icosiella neglecta* in water frogs of western Palearctic region [31] showed an overall haplotype diversity comparable to our (Hd = 0.729 in *I*. *neglecta* vs Hd = 0.702 in *P*. *oryctolagi*) but only a weak population-genetic structure. Besides host dispersal behaviour, other factors should be considered to explain the differences in population structure between *I*. *neglecta* and *P*. *oryctolagi*. In the first case, the parasite is a vector-transmitted species with a relatively wide spectrum of hosts. Conversely, *P*. *oryctolagi* is more species-specific and does not require a vector for transmission. These factors, along with the small home range of brown hares [32], promote the presence of genetic structure among samples of *P*. *oryctolagi* from different geographical areas, even at relatively low geographical scales. The gastropod intermediate host might play a role too in shaping the observed genetic diversity. In France are reported as possible intermediate host three genera belonging to Hygromiidae family [4], while no data are available in the Italian territory.

### Genetic variability between lineages A and B in *P*. *oryctolagi* of northern and central Italy

The 3% sequence variability between the two *P*. *oryctologi* mitotype-clusters A and B is sometimes similar to, exceeds, or is smaller if compared to the intraspecific variability computed for the *cox1*-bacode sequences of several genera of Nematoda [27,33]. Two evolutionary scenarios can be hypothesized to explain the observed intraspecific variation: the first and more conventional one implies that all sequenced specimens, irrespective to their mitotype, belong to the single species *P*. *oryctolagi*. This scenario is supported by the absence of evident morphological differences among the specimens assigned to the same morpho-species based on traditional diagnostic characters. Moreover, the 3% value of intraspecific variability, although notable, is not an extreme value for Nematoda [27]. A second more intriguing conceivable evolutionary scenario suggests that nematodes sharing mitotypes A and B form two distinct phyletic groups that are evolving independently. If this second hypothesis, *P*. *oryctoloagi* should be considered as a complex of species. Supporting this alternative scenario, the two groups of mitotypes are distinctly separated with no intermediate haplotypes identified. Lack of morphological differentiation could be real, as expected in early stages of speciation, or due to the overlooking of some small diagnostic features not detectable with the traditional optical microscopy. DNA-barcode sequences alone cannot resolve this issue. A comprehensive solution requires additional molecular markers both mitochondrial (complete genomes) and nuclear as well as a thorough morphological/anatomical investigation using the more recent and sophisticated microscopy approaches.

### Geographic distribution of mitotypes and presence of barriers to gene flow

Lineages A and B appear not randomly distributed, with lineage A prevalent in Central Italy and lineage B exclusive of northern areas. The high level of genetic differences between the lineages A and B and their geographical distribution could have two alternative explanations. They may (i) reflect the natural history of colonization of their hosts from Eastern Europe towards central western territories during late Pleistocene [34]; or, as alternative hypothesis, (ii) they have occurred in recent times because of the massive release of exotic lineages for hunting purposes. This practice, started in the early 1900s, peaked in 1970–1990 with an annual release of 90,000–120,000 units coming mainly from East Europe [35]. In both hypotheses -past spontaneous migration or relatively recent translocations- these two events would have resulted in the dilution of the autochthonous parasite strain (that is the A-group mitotypes) with exotic lineages both of hosts and parasites (i.e. B-group mitotypes). In particular, the B-group individuals could belong in the first hypothesis to an eastern strain that migrated passively with their definitive hosts during the last glacial period or, in the second hypothesis, to exotic lineages recently translocated for hunting purposes. Human activity may also explain the presence of the few individuals of lineage A found outside its main geographical range.

The observed non-random distribution of the 11 mitotypes is statistically supported by the AMOVA analysis, which identified the presence of a strong main barrier to gene flow which separates samples of *P*. *oryctolagi* of Northern Italy from those of Central Italy, with high and highly significative Φ_ST_. From a strictly geographic point of view this barrier is delineated by the presence of the Tuscan-Emilian Apennine ridge. A second minor yet noteworthy barrier separates individuals of Pianosa island from those from Grosseto, with a low but significant Φ_ST_. The Pianosa group represents an unusual sample: despite being the largest one (Table 1), it presents only two haplotypes. Nineteen out of 21 individuals share the most frequent A1 haplotype typical of Central Italy, while the remaining two exhibit the A-derived A3 private haplotype, exclusive of Pianosa. This scarce diversity could be attributed to a genetic bottleneck due to founder effect occurred in recent times. This hypothesis is supported by two different historical documents both reporting that brown hares were introduced on the island by humans for hunting purposes in the middle 1800s [36,37], about 50 years before the translocation of the first hares from East Europe. On the contrary, no genetic signs of possible “environmental effect” were detected among northern samples coming from locations with very different agricultural pressure, i.e. the Po plain (RE1 and MO1, characterized by extreme habitat homogeneity and anthropization), and hill (RE3, with higher habitat diversity and availability of shelters).

## Conclusions

This genetic survey, based on *cox1*-barcode sequences and the related frequency and distribution over space, revealed the presence of two distinct populations of *P*. *oryctolagi* across five areas of northern and central Italy. Considering the Tuscan-Emilian Apennine ridge as a possible natural geographical boundary, one population appears confined to the north, while the other is primarily found in samples south of the Apennines, although a few individuals of this latter population were found also in northern territories. The main drivers that likely influenced the population structure observed in *P*. *oryctolagi* of Northern and Central Italy are the small home range of its hosts and the biology of the parasite itself, which shows a relatively narrow spectrum of hosts. Alongside these factors, natural dispersion and passive human-mediated migration should also be considered. Human activity linked to translocations for hunting purposes may also explain the presence of a slight but detectable divergence among samples from Central Italy (Pianosa and Grosseto), whereas human activity due to agriculture seems not to impact with the population structure of *P*. *oryctolagi*. The significant level of intraspecific genetic differentiation leads us to propose alternative micro-evolutionary scenarios for *P*. *oryctolagi* which require further analyses for verification.

## Supporting information

S1 Fig Multiple alignment of the different unique *cox1* mito-haplotypes of *Protostrongylus oryctolagi*. (PDF)

S2 Fig Pairwise alignment of NUMTs of *Protostrongylus oryctolagi* L15_1_Modena and L19_1_female_Modena. (PDF)

S3 Fig Pairwise alignment of *cox1* and NUMT of of *Protostrongylus oryctolagi* L15_1_Modena and translation of the NUMT in polypeptides. (PDF)

S4 Fig Pairwise alignment of *cox1* and NUMT of *Protostrongylus oryctolagi* L19_1_female_Modena and translation of the NUMT in polypeptides. (PDF)

S1 Table P-distances among unique mitotypes of *Protostrongylus oryctolagi*. (PDF)
