# Supplementary material for: Genetic variability and population structure analysis of Protostrongylus oryctolagi (Nematoda: Protostrongylidae) in Lepus europaeus from Central and Northern Italy
Source: PLoS One. 2025 Jan 9;20(1):e0313998. doi: 10.1371/journal.pone.0313998 (PMC11717190; doi:10.1371/journal.pone.0313998)
Supplement: S1 Fig — (PDF) [file pone.0313998.s001.pdf]

S1 Figure. Multiple alignment of the different unique *cox1* mito-haplotypes of *Protostrongylus oryctolagi*.

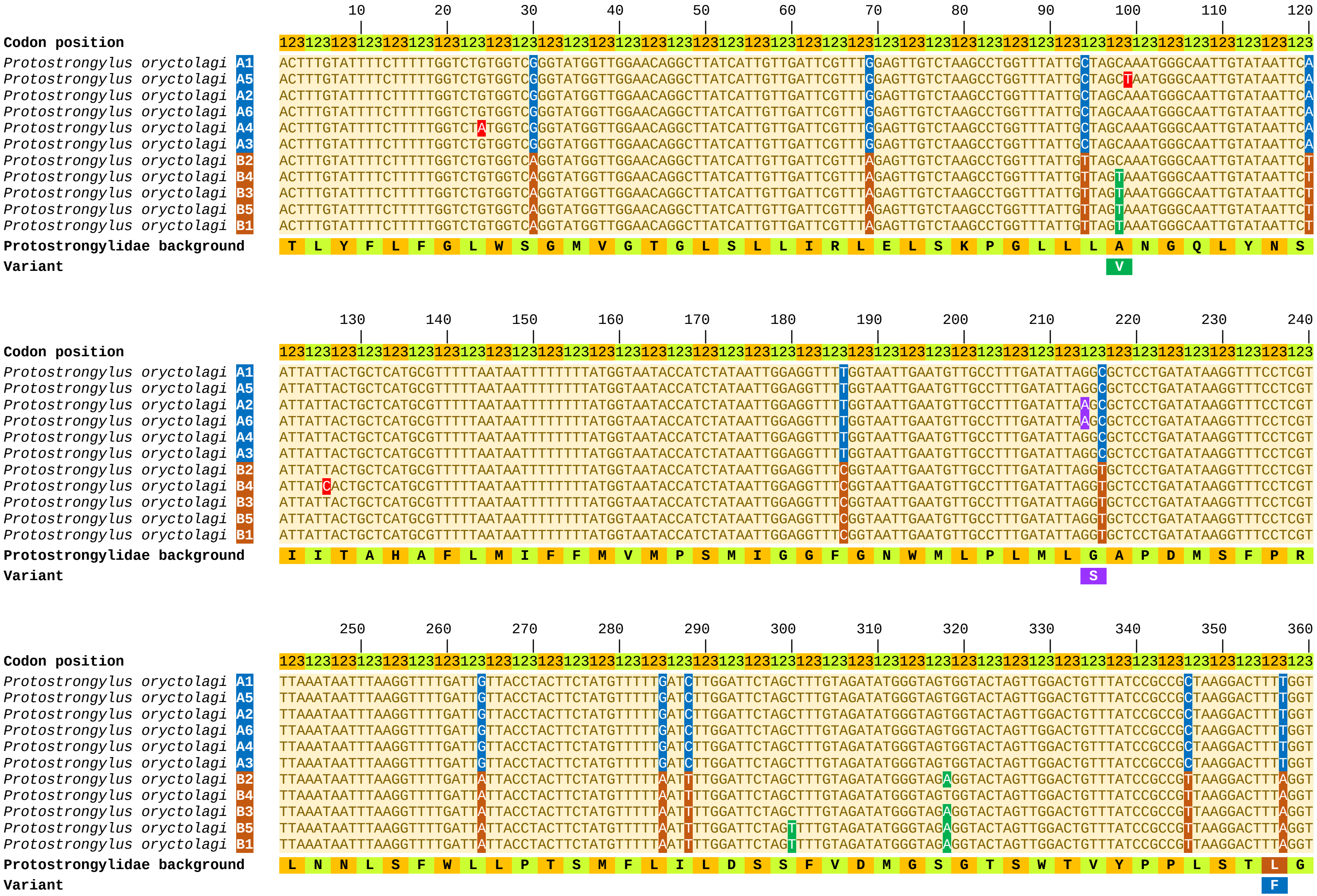

S1 Figure. Multiple alignment of the different unique *cox1* mito-haplotypes of *Protostrongylus oryctolagi*.


```
                                  370       380       390       400       410       420       430       440       450       460       470       480
Codon position                 123123123123123123123123123123123123123123123123123123123123123123123123123123123123123123123123123123123123123123123123
Protostrongylus oryctolagi A1  CATGTTGGTAGAAGGGTTGATTTAGCTATTTTTAGTTTGCATTGTGCTGGGTTAAGATCGATTTTGGGTGGAATTAATTTTATGTGTACTACTAAAAATATGCGAAGGAGGTCTATTTCA
Protostrongylus oryctolagi A5  CATGTTGGTAGAAGGGTTGATTTAGCTATTTTTAGTTTGCATTGTGCTGGGTTAAGATCGATTTTGGGTGGAATTAATTTTATGTGTACTACTAAAAATATGCGAAGGAGGTCTATTTCA
Protostrongylus oryctolagi A2  CATGTTGGTAGAAGGGTTGATTTAGCTATTTTTAGTTTGCATTGTGCTGGGTTAAGATCGATTTTGGGTGGAATTAATTTTATGTGTACTACTAAAAATATGCGAAGGAGGTCTATTTCA
Protostrongylus oryctolagi A6  CATGTTGGTAGAAGGGTTGATTTAGCTATTTTTAGTTTGCATTGTGCTGGGTTAAGATCGATTTTGGGTGGAATTAATTTTATGTGTACTACTAAAAATATGCGAAGGAGGTCTATTTCA
Protostrongylus oryctolagi A4  CATGTTGGTAGAAGGGTTGATTTAGCTATTTTTAGTTTGCATTGTGCTGGGTTAAGATCGATTTTGGGTGGAATTAATTTTATGTGTACTACTAAAAATATGCGAAGGAGGTCTATTTCA
Protostrongylus oryctolagi A3  CATGTTGGTAGAAGGGTTGATTTAGCTATTTTTAGTTTGCATTGTGCTGGGTTAAGATCGATTTTGGGTGGAATTAATTTTATGTGTACTACTAAAAATATGCGAAGGAGGTCTATTTCA
Protostrongylus oryctolagi B2  CATGTAGGTAGAAGGGTTGATTTAGCTATTTTTAGTTTACATTGTGCTGGGTTAAGGTCGATTTTGGGTGGAATTAATTTTATGTGTACTACTAAAAATATGCGGAGGAGGTCTATTTCA
Protostrongylus oryctolagi B4  CATGTAGGTAGAAGGGTTGATTTAGCTATTTTTAGTTTACATTGTGCTGGGTTAAGGTCGATTTTGGGTGGAATTAATTTTATGTGTACTACTAAAAATATGCGGAGGAGGTCTATTTCA
Protostrongylus oryctolagi B3  CATGTAGGTAGAAGGGTTGATTTAGCTATTTTTAGTTTACATTGTGCTGGGTTAAGGTCGATTTTGGGTGGAATTAATTTTATGTGTACTACTAAAAATATGCGGAGGAGGTCTATTTCA
Protostrongylus oryctolagi B5  CATGTAGGTAGAAGGGTTGATTTAGCTATTTTTAGTTTACATTGTGCTGGGTTAAGGTCGATTTTGGGTGGAATTAATTTTATGTGTACTACTAAAAATATGCGGAGGAGGTCTATTTCA
Protostrongylus oryctolagi B1  CATGTAGGTAGAAGGGTTGATTTAGCTATTTTTAGTTTACATTGTGCTGGGTTAAGGTCGATTTTGGGTGGAATTAATTTTATGTGTACTACTAAAAATATGCGGAGGAGGTCTATTTCA
Protostrongylidae background     H  V  G  S  S  V  D  L  A  I  F  S  L  H  C  A  G  L  S  S  I  L  G  G  I  N  F  M  C  T  T  K  N  M  R  S  S  S  I  S
Variant
```

```
                                  490       500       510       520       530       540       550       560       570       580       590       600
Codon position                 123123123123123123123123123123123123123123123123123123123123123123123123123123123123123123123123123123123123123123123123
Protostrongylus oryctolagi A1  TTGGAGCATATAAGGTTATTTGTTTGAGCGGTTTTTGTTACTGTTTTTTTTATTAGTATTGTCTTTACCAGTGTTAGCTGGTGCTATTACTATATTATTAACAGATCGTAATTTAAATACT
Protostrongylus oryctolagi A5  TTGGAGCATATAAGGTTATTTGTTTGAGCGGTTTTTGTTACTGTTTTTTTTATTAGTATTGTCTTTACCAGTGTTAGCTGGTGCTATTACTATATTATTAACAGATCGTAATTTAAATACT
Protostrongylus oryctolagi A2  TTGGAGCATATAAGGTTATTTGTTTGAGCGGTTTTTGTTACTGTTTTTTTTATTAGTATTGTCTTTACCAGTGTTAGCTGGTGCTATTACTATATTATTAACAGATCGTAATTTAAATACT
Protostrongylus oryctolagi A6  TTGGAGCATATAAGGTTATTTGTTTGAGCGGTTTTTGTTACTGTTTTTTTTATTAGTATTGTCTTTACCAGTGTTAGCTGGTGCTATTACTATATTATTAACAGATCGTAATTTAAATACC
Protostrongylus oryctolagi A4  TTGGAGCATATAAGGTTATTTGTTTGAGCGGTTTTTGTTACTGTTTTTTTTATTAGTGTTGTCTTTACCAGTGTTAGCTGGTGCTATTACTATATTATTAACAGATCGTAATTTAAATACT
Protostrongylus oryctolagi A3  TTGGAGCATATAAGGTTATTTGTTTGAGCGGTTTTTGTTACTGTTTTTTTTATTAGTATTGTCTTTACCAGTGTTAGCTGGTGCTATTACTATATTATTAACAGATCGTAATTTAAATACT
Protostrongylus oryctolagi B2  TTGGAGCATATAAGGTTATTTGTTTGAGCGGTTTTTGTTACTGTTTTTTTTATTAGTATTGTCTTTACCAGTGTTAGCTGGTGCTATTACTATATTATTAACAGATCGTAATTTGAATACT
Protostrongylus oryctolagi B4  TTGGAGCATATAAGGTTATTTGTTTGAGCGGTTTTTGTTACTGTTTTTTTTATTAGTATTGTCTTTACCAGTGTTAGCTGGTGCTATTACTATATTATTAACAGATCGTAATTTGAATACT
Protostrongylus oryctolagi B3  TTGGAGCATATAAGGTTATTTGTTTGAGCGGTTTTTGTTACTGTTTTTTTTATTAGTATTGTCTTTACCAGTGTTAGCTGGTGCTATTACTATATTATTAACAGATCGTAATTTGAATACT
Protostrongylus oryctolagi B5  TTGGAGCATATAAGGTTATTTGTTTGAGCGGTTTTTGTTACTGTTTTTTTTATTAGTATTGTCTTTACCAGTGTTAGCTGGTGCTATTACTATATTATTAACAGATCGTAATTTAAATACT
Protostrongylus oryctolagi B1  TTGGAGCATATAAGGTTATTTGTTTGAGCGGTTTTTGTTACTGTTTTTTTTATTAGTATTGTCTTTACCAGTGTTAGCTGGTGCTATTACTATATTATTAACAGATCGTAATTTGAATACT
Protostrongylidae background     L  E  H  M  S  L  F  V  W  A  V  F  V  T  V  F  L  L  V  L  S  L  P  V  L  A  G  A  I  T  M  L  L  T  D  R  N  L  N  T
Variant
```

```
                                  610       620       630       640
Codon position                 123123123123123123123123123123123123123123123123
Protostrongylus oryctolagi A1  TCTTTTTTTGATCCTAGTTCTGGGGGTAATCCTTTAATTTATCAACAT
Protostrongylus oryctolagi A5  TCTTTTTTTGATCCTAGTTCTGGGGGTAATCCTTTAATTTATCAACAT
Protostrongylus oryctolagi A2  TCTTTTTTTGATCCTAGTTCTGGGGGTAATCCTTTAATTTATCAACAT
Protostrongylus oryctolagi A6  TCTTTTTTTGATCCTAGTTCTGGGGGTAATCCTTTAATTTATCAACAT
Protostrongylus oryctolagi A4  TCTTTTTTTGATCCTAGTTCTGGGGGTAATCCTTTAATTTATCAACAT
Protostrongylus oryctolagi A3  TCTTTTTTTGATCCTAGTTCTGGAGGTAATCCTTTAATTTATCAACAT
Protostrongylus oryctolagi B2  TCTTTTTTTGATCCTAGTTCTGGAGGTAATCCTTTAATTTATCAACAT
Protostrongylus oryctolagi B4  TCTTTTTTTGATCCTAGTTCTGGAGGTAATCCTTTAATTTATCAACAT
Protostrongylus oryctolagi B3  TCTTTTTTTGATCCTAGTTCTGGAGGTAATCCTTTAATTTATCAACAT
Protostrongylus oryctolagi B5  TCTTTTTTTGATCCTAGTTCTGGGGGTAATCCTTTAATTTATCAACAT
Protostrongylus oryctolagi B1  TCTTTTTTTGATCCTAGTTCTGGAGGTAATCCTTTAATTTATCAACAT
Protostrongylidae background     S  F  F  D  P  S  S  G  G  N  P  L  I  Y  Q  H
Variant
```

X, nucleotide conserved in the majority of mitotypes. X, nucleotide conserved in mitotypes A. X, nucleotide conserved in mitotypes B. X, different nucleotide restricted to a single sequence. X, variant nucleotide shared by some mitotypes A. X, variant nucleotide shared by some mitotypes B. X, variant nucleotide shared by some mitotypes A-B.
123 / 123, positions 1-3 in the codon. The portion of the protein encoded is figured on the bottom with the variant amino acids included.
Alignment length: 648 positions. Identical positions: 622 (95.99 %). Variable positions 26 (4.01%).
