# Supplementary material for: Genetic variability and population structure analysis of Protostrongylus oryctolagi (Nematoda: Protostrongylidae) in Lepus europaeus from Central and Northern Italy
Source: PLoS One. 2025 Jan 9;20(1):e0313998. doi: 10.1371/journal.pone.0313998 (PMC11717190; doi:10.1371/journal.pone.0313998)
Supplement: S2 Fig — (PDF) [file pone.0313998.s002.pdf]

S2 Figure. Pairwise alignment of **NUMTs** of *Protostrongylus oryctolagi* L15_1_Modena and L19_1_female_Modena.

```
                                 10         20         30         40         50         60         70         80         90        100        110        120
                                  |          |          |          |          |          |          |          |          |          |          |          |
L15_1_Modena NUMT         TTTTAGTTTGTGATCAGGTATGGTAGGGACGGGAATATCTTTGTCAATTCGTTCGGAGCTATCTAAACTGGGTTTATTATTGGCCATTGGTCACTTATAAAGTTAAATTATTACAGTACA
L19_1_female_Modena NUMT  TTTTAGTTTGTGATCAGGTATGGTAGGGACGGGAATATCTTTGTCAATTCGTTCGGAGCTATCTAAACTGGGTTTATTATTGGCCATTGGTCACTTATAAAGTTAAATTATTACAGTACA

                                130        140        150        160        170        180        190        200        210        220        230        240
                                  |          |          |          |          |          |          |          |          |          |          |          |
L15_1_Modena NUMT         TGGGTACATTTTTATGGTAATACCTTCTATAATTGGAAGTTTGGTACTGGATATTACCTCTAATGTTAGGTGCTTTAAATAATTTGAGATTTTGGTTGTTATCTACTTCTGTGTTTTTGA
L19_1_female_Modena NUMT  TGGGTACATTTTTATGGTAATACCTTCTATAATTGGAAGTTTGGTACTGGATATTACCTCTAATGTTAGGTGCTTTAAATAATTTGAGATTTTGGTTGTTATCTACTTCTGTGTTTTTGA

                                250        260        270        280        290        300        310        320        330        340        350        360
                                  |          |          |          |          |          |          |          |          |          |          |          |
L15_1_Modena NUMT         TTTTGGACTCTAGGACAATATAGGAAGTGGTACTAGTTGGACTGTGTATCCACCTTTAAGTACTTAGGGTCATGTGGGTAGTTAGATTTAGCTGTTTTTAGTTTGCACTGTGCTAGTTTG
L19_1_female_Modena NUMT  TTTTGGACTCTGGGACAATATAGGAAGTGGTACTAGTTGGACTGTGTATCCACCTTTAAGTACTTAGGGTCATGTGGGTAGTTAGATTTAGCTGTTTTTAGTTTGCACTGTGCTAGTTTG

                                370        380        390        400        410        420        430        440        450        460        470        480
                                  |          |          |          |          |          |          |          |          |          |          |          |
L15_1_Modena NUMT         AGGTCTACTTTGGGAGGTATTAATTTTATGTGTACTACAAAAGATATACTTAGGTCTGGTTCTTTGGAACATATAAGGTTGTTTGTTTGGGCGGTTTTTGTTACTGTTCTTTTGTCACTT
L19_1_female_Modena NUMT  AGGTCTACTTTGGGAGGTATTAATTTTATGTGTACTACAAAAGATATACTTAGGTCTGGTTCTTTGGAACATATAAGGTTGTTTGTTTGGGCGGTTTTTGTTACTGTTCTTTTGTCACTT

                                490        500        510        520        530        540        550        560        570        580        590        600
                                  |          |          |          |          |          |          |          |          |          |          |          |
L15_1_Modena NUMT         TTGTCGTTATCAGTGTTGGCTGGTGCAATTACAATGCTATTAATTGATATTAATTCAAATACTTCTTTTTTTGATCCTATTTCTGGAGGCATTCCTTTAGTCTATCAACATTTATTT
L19_1_female_Modena NUMT  TTGTCGTTATCAGTGTTGGCTGGTGCAATTACAATGCTATTAATTGATATTAATTCAAATACTTCTTTTTTTGATCCTATTTCTGGAGGCATTCCTTTAGTCTATCAACATTTATTT
```
