# Supplementary figures and images for: Genetic variability and population structure analysis of Protostrongylus oryctolagi (Nematoda: Protostrongylidae) in Lepus europaeus from Central and Northern Italy
Source: PLoS One. 2025 Jan 9;20(1):e0313998. doi: 10.1371/journal.pone.0313998 (PMC11717190; doi:10.1371/journal.pone.0313998)

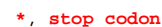

Supplement: S3 Fig — (PDF) [file pone.0313998.s003.pdf]
