# Supplementary material for: Genetic variability and population structure analysis of Protostrongylus oryctolagi (Nematoda: Protostrongylidae) in Lepus europaeus from Central and Northern Italy
Source: PLoS One. 2025 Jan 9;20(1):e0313998. doi: 10.1371/journal.pone.0313998 (PMC11717190; doi:10.1371/journal.pone.0313998)
Supplement: S4 Fig — (PDF) [file pone.0313998.s004.pdf]

S4 Figure. Pairwise alignment of ***cox1*** and **NUMT** of *Protostrongylus oryctolagi* L19_1_female_Modena and translation of the **NUMT** in polypeptides.

```
                                  10        20        30        40        50        60        70        80        90       100       110       120
                                   |         |         |         |         |         |         |         |         |         |         |         |
L19_1_female_Modena cox1   ACTTTGTATTTTCTTTTTGGTCTGTGGTCAGGTATGGTTGGAACAGGCTTATCATTGTTGATTCGTTTAGAGTTGTCTAAGCCTGGTTTATTGTTAGTAAATGGGCAATTGTATAATTCT
L19_1_female_Modena NUMT   --------------TTTTAGTTTGTGATCAGGTATGGTAGGGACGGGAATATCTTTGTCAATTCGTTCGGAGCTATCTAAACTGGGTTTATTATTGGCCATTGGTCACTTATAAAGTTAA

                                 130       140       150       160       170       180       190       200       210       220       230       240
                                   |         |         |         |         |         |         |         |         |         |         |         |
L19_1_female_Modena cox1   ATTATTACTGCTCATGCGTTTTTAATAATTTTTTTTATGGTAATACCATCTATAATTGGAGGTTTCGGTAATTGAATGTTGCCTTTGATATTAGGTGCTCCTGATATAAGGTTTCCTCGT
L19_1_female_Modena NUMT   ATTATTACAGTACATG-GGT----ACA----TTTTTATGGTAATACCTTCTATAATTGGAAGTTT-GGTA-CTGGATATTACCTCTAATGTTAGGTGCT--------------------

                                 250       260       270       280       290       300       310       320       330       340       350       360
                                   |         |         |         |         |         |         |         |         |         |         |         |
L19_1_female_Modena cox1   TTAAATAATTTAAGGTTTTGATTATTACCTACTTCTATGTTTTTAATTTTGGATTCTAGTTTTGTAGATATGGGTAGAGGTACTAGTTGGACTGTTTATCCGCCGTTAAGGACTTTAGGT
L19_1_female_Modena NUMT   TTAAATAATTTGAGATTTTGGTTGTTATCTACTTCTGTGTTTTTGATTTTGGACTCTGG----GACAATATAGGAAGTGGTACTAGTTGGACTGTGTATCCACCTTTAAGTACTTAGGGT

                                 370       380       390       400       410       420       430       440       450       460       470       480
                                   |         |         |         |         |         |         |         |         |         |         |         |
L19_1_female_Modena cox1   CATGTAGGTAGAAGGGTTGATTTAGCTATTTTTAGTTTACATTGTGCTGGGTTAAGGTCGATTTTGGGTGGAATTAATTTTATGTGTACTACTAAAAATATGCGGAGGAGGTCTATTTCA
L19_1_female_Modena NUMT   CATGTGGGTAG----TTAGATTTAGCTGTTTTTAGTTTGCACTGTGCTAGTTTGAGGTCTACTTTGGGAGGTATTAATTTTATGTGTACTACAAAAGATATACTT---AGGTCTGGTTCT

                                 490       500       510       520       530       540       550       560       570       580       590       600
                                   |         |         |         |         |         |         |         |         |         |         |         |
L19_1_female_Modena cox1   TTGGAGCATATAAGGTTATTTGTTTGAGCGGTTTTTGTTACTGTTTTTTTATTAGTATTGTCTTTACCAGTGTTAGCTGGTGCTATTACTATATTATTAACAGATCGTAATTTGAATACT
L19_1_female_Modena NUMT   TTGGAACATATAAGGTTGTTTGTTTGGGCGGTTTTTGTTACTGTTCTTTTGTCACTTTGTCGTTATCAGTGTTGGCTGGTGCAATTACAATGCTATTAATTTGATATTAATTCAAATACT

                                 610       620       630       640
                                   |         |         |         |
L19_1_female_Modena cox1   TCTTTTTTTGATCCTAGTTCTGGAGGTAATCCTTTAATTTATCAACAT
L19_1_female_Modena NUMT   TCTTTTTTTGATCCTATTTCTGGAGGCATTCCTTTAGTCTATCAACAT
```

Alignment length: 648. Identity: 479 (73.92%). Different: 169 (26.08%).

```
L19_1_female_Modena cox1 (reading frame 1, translation)
TLYFLFGLWSGMVGTGLSLLIRLELSKPGLLLVNGQLYNSIITAHAFLMIFFMVMPSMIGGFGNWMLPLMLGAPDMSFPR
LNNLSFWLLPTSMFLILDSSFVDMGSGTSWTVYPPLSTLGHVGSSVDLAIFSLHCAGLSSILGGINFMCTTKNMRSSSIS
LEHMSLFVWAVFVTVFLLVLSLPVLAGAITMLLTDRNLNTSFFDPSSGGNPLIYQH

L19_1_female_Modena NUMT (reading frame 1, translation)
F*FVISYGSDGNIFVNSFGAI*TGFIIGHWSLMKLNYYSTWVHFYGNTFYNWKFGTGYYL*C*VL*MIWDFGCYLLLCFW
FWTLGQYSKWY*LDCVSTFKYLGSCG*LDLAVFSLHCASLSSTLGGINFMCTTKDMLSSGSLEHMSLFVWAVFVTVLLSL
LSLSVLAGAITMLLIDINSNTSFFDPISGGIPLVYQH

L19_1_female_Modena NUMT (reading frame 2, translation)
FSLWSGMVGTGMSLSIRSELSKLGLLLAIGHL*S*IITVHGYIFMVMPSMIGSLVLDITSNVSCFK*FEILVVIYFCVFD
FGLWDNMGSGTSWTVYPPLST*GHVGS*I*LFLVCTVLVWGLLWEVLILCVLQKMYLGLVLWNM*GCLFGRFLLLFFCHF
CRYQCWLVQLQCY*LMLIQMLLFLILFLEAFL*SINIY

L19_1_female_Modena NUMT (reading frame 3, translation)
LVCDQVW*GREYLCQFVRSYLNWVYYWPLVTYKVKLLQYMGTFLW*YLL*LEVWYWMLPLMLGALNNLSFWLLSTSVFLI
LDSGTM*EVVLVGLCIHL*VLSVMWVVSFSCF*FALC*FEVYFGSY*FYVYYKSYT*VWFFGTYKVVCLGGFCYCSFVTF
VVISVGWCNYNAINWY*FKYFFFWSYFWSHSFSLSTFI
```

***, stop codon**
