# Supplementary material for: Genetic variability and population structure analysis of Protostrongylus oryctolagi (Nematoda: Protostrongylidae) in Lepus europaeus from Central and Northern Italy
Source: PLoS One. 2025 Jan 9;20(1):e0313998. doi: 10.1371/journal.pone.0313998 (PMC11717190; doi:10.1371/journal.pone.0313998)
Supplement: S1 Table — (PDF) [file pone.0313998.s005.pdf]

S1 Table. P-distances among unique mitotypes of *Protostrongylus oryctolagi.*

p-distances

	MIT	A1	A2	A3	A4	A5	A6	B1	B2	B3	B4	B5
Protostrongylus oryctolagi	A1											
Protostrongylus oryctolagi	A2	**0.002**										
Protostrongylus oryctolagi	A3	**0.002**	0.003									
Protostrongylus oryctolagi	A4	0.003	0.005	0.005								
Protostrongylus oryctolagi	A5	**0.002**	0.003	0.003	0.005							
Protostrongylus oryctolagi	A6	0.003	**0.002**	0.005	**0.006**	0.005						
Protostrongylus oryctolagi	B1	0.031	0.032	0.029	**0.034**	0.032	**0.034**					
Protostrongylus oryctolagi	B2	0.028	0.029	**0.026**	0.031	0.029	0.031	0.003				
Protostrongylus oryctolagi	B3	0.029	0.031	0.028	0.032	0.031	0.032	**0.002**	**0.002**			
Protostrongylus oryctolagi	B4	0.029	0.031	0.028	0.032	0.031	0.032	0.005	0.005	0.003		
Protostrongylus oryctolagi	B5	0.028	0.029	0.029	0.031	0.029	0.031	0.003	0.006	0.005	**0.008**	

0.003	0.001
avg	std

0.030	0.002
avg	std

0.004	0.002
avg	std

Number of different nucleotides

	MIT	A1	A2	A3	A4	A5	A6	B1	B2	B3	B4	B5
Protostrongylus oryctolagi	A1											
Protostrongylus oryctolagi	A2	**1**										
Protostrongylus oryctolagi	A3	**1**	2									
Protostrongylus oryctolagi	A4	2	3	3								
Protostrongylus oryctolagi	A5	**1**	2	2	3							
Protostrongylus oryctolagi	A6	2	**1**	3	**4**	3						
Protostrongylus oryctolagi	B1	20	21	19	**22**	21	**22**					
Protostrongylus oryctolagi	B2	18	19	**17**	20	19	20	2				
Protostrongylus oryctolagi	B3	19	20	18	21	20	21	**1**	**1**			
Protostrongylus oryctolagi	B4	19	20	18	21	20	21	3	3	2		
Protostrongylus oryctolagi	B5	18	19	19	20	19	20	2	4	3	**5**	

2.20	0.941
avg	std

19.7	1.236
avg	std

3	1.265
avg	std

MIT = MITotype
avg = average
std = standard deviation
